# Truncated TPPP – An Endopterygota-specific protein

**DOI:** 10.1016/j.heliyon.2021.e07135

**Published:** 2021-05-24

**Authors:** Ferenc Orosz

**Affiliations:** Institute of Enzymology, Research Centre for Natural Sciences, Magyar Tudósok körútja 2, 1117 Budapest, Hungary

**Keywords:** Endopterygota, Lepidoptera, Phylogenetic tree, p25alpha domain

## Abstract

TPPP proteins exhibiting microtubule stabilizing function constitute a eukaryotic protein superfamily, characterized by the presence of the p25alpha domain of various lengths. Vertebrate species possess three TPPP paralogs; all of them possess a full-length p25alpha domain of 160–170 amino acids and are encoded by three exons. Species of Endopterygota (Holometabola) have, besides a full-size TPPP ortholog, a protein with a truncated p25alpha domain as well, where the last coding exon, responsible for microtubule binding, is missing. It is not the result of an alternative splicing but is coded by another gene. In *Drosophila melanogaster*, they are named as CG45057 (long-type) and CG6709 (truncated). The truncated protein has been found in the Endopterygota orders Diptera, Coleoptera, Hymenoptera, Lepidoptera and Raphidioptera. In Lepidoptera, in several superfamilies (Gelechioidea, Bombycoidea, Noctuoidea, Pyraloidea) two paralogs of the truncated TPPP occur. Truncated orthologs (CG6709) were not found in other insects or in arthropods and are absent in any other organism, as well, while the long-type TPPPs (CG45057 orthologs) occur commonly in all animals. Thus it seems that CG6709 orthologs occur only in insects undergoing on metamorphosis.

## 1 Introduction

TPPP-like proteins constitute a eukaryotic protein superfamily, characterized by the presence of the p25alpha domain (PF05517, IPR008907) [1]. TPPP-like proteins are named after the first identified member, Tubulin Polymerization Promoting Protein, TPPP/p25 or TPPP1, exhibiting microtubule stabilizing function [2, 3]. This function seems to be conserved in animals from sponge to vertebrates [4]; it is also true for TPPP of *Drosophila melanogaster*, CG45057 [5]. TPPPs, in the strict sense, contain no other domains but a p25alpha domain of various lengths. Vertebrate species possess generally three TPPP paralogs [6], more precisely, outparalogs [7], i.e., genes/proteins, in the *same* species, diverged from a common ancestral TPPP as a consequence of genetic duplication in a common ancestor of vertebrates; all of them possess a full-length p25alpha domain of 160–170 amino acids (aa) and are encoded by three exons [8]. Other animals have generally only one TPPP ortholog (orthologs are genes in *different* species evolved from a common ancestral gene), also with a full-length p25 domain. The number of the coding exons is also three in most cases; however, in some insects, as in various *Drosophila* species and in *Tribolium castaneum*, the first two exons are merged. Preliminary BLAST search indicated that some species of the phylum Arthropoda contain an additional TPPP-like protein. Here I show that they are present only in insects which undergo complete metamorphosis (Endopterygota or Holometabola).

## 2 Methods

BLASTP and TBLASTN analyses [9] were performed on protein and nucleotide sequences available at the NCBI website, http://www.ncbi.nlm.nih.gov/BLAST/ using *D. melanogaster* NP_648370 protein as a query. If a hit was found in a species of a given phylogenetic branch then its sequence was used as a query within the whole branch. All the protein and nucleotide databases available at this webpage were searched. Nucleotide sequences identified in BLASTN searches were translated in the reading frames denoted in the BLASTN hit, taking frame shifts or introns of genomic sequences into account. Further BLAST search was carried at the Ensemble website, http://metazoa.ensembl.org/, where few additional hits were found. Accession numbers of protein and nucleotide sequences refer to the NCBI GenBank database except otherwise stated.

### 2.1 Phylogenetic analysis

Multiple alignments of sequences were carried out by the Clustal Omega program [10]. Multiple sequence alignments used for constructing phylogenetic trees are shown in Supplementary Figures 1 and 2. The MEGA5 software [11] was used for maximum parsimony (MP) analysis. The MP tree was obtained using the Close-Neighbor-Interchange algorithm [12] in which the initial trees were obtained with the random addition of sequences (10 replicates). A majority-rule consensus tree was generated from the equally most parsimonious trees using the Consensus Tree option of the program. Internal support was assessed by non-parametric bootstrapping [13]; parsimony bootstrap percentages were based on 1000 replicates. Gaps were treated as missing data. Bayesian analysis was performed using MrBayes v3.1.2 [14]. Default priors and the WAG model [15] were used assuming equal rates across sites. Two independent analyses were run with three heated and one cold chains (temperature parameter 0.2) for 3 × 10^6^ generations, with a sampling frequency of 0.01 and the first 25 % of the generations were discarded as burn-in. The two runs were convergent.

## 3 Results

**Figure 1:**
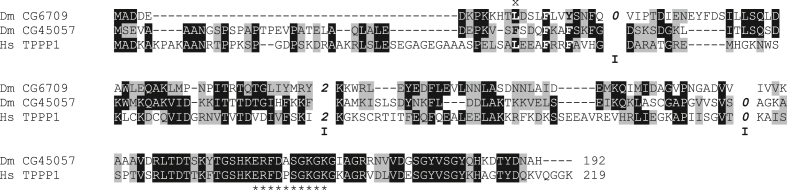
Alignment used for sequence similarity comparisons of human TPPP1 and *Drosophila melanogaster* TPPP proteins. Intron positions were added manually in front of the affected amino acid position. The intron boundaries are indicated, also giving their phase. Asterisks show the tubulin binding sequence; x denotes the beginning of the p25alpha domain. DmCG6709 - *Drosophila melanogaster* NP_648370; DmCG45057 - *Drosophila melanogaster* NP_648881; Hs – *Homo sapiens* NP_008961.

**Figure 2:**
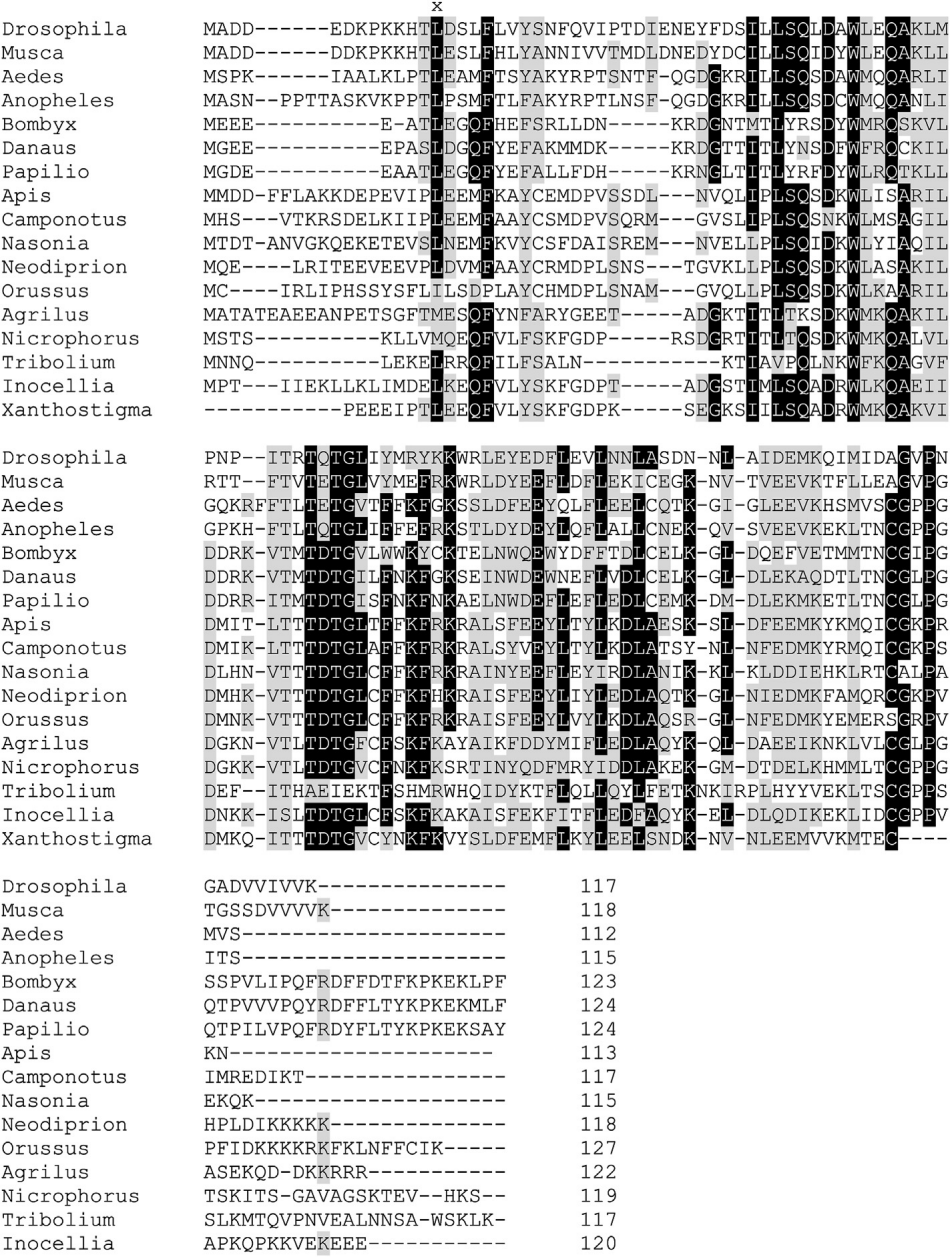
Multiple alignment of Endopterygota CG6709 orthologs obtained by Clustal Omega program [12]. The alignment was refined manually. “x” denotes the beginning of the p25alpha domain. Residues identical and similar in the majority of the species are indicated by black and grey backgrounds, respectively. Diptera, Brachycera: *Drosophila melanogaster* NP_648370, *Musca domestica* XP_005179904; Diptera, Nematocera: *Aedes albopictus* XP_019531996, *Anopheles gambiae* XP_556944; Lepidoptera: *Bombyx mori* XP_004933177, *Danaus plexippus* XP_032527880, *Papilio polytes* XP_013136556; Hymenoptera, Apocrita: *Apis dorsata* XP_006607661, *Camponotus floridanus* XP_011254991, *Nasonia vitripennis* XP_008211062; Hymenoptera, Tenthredinoidea: *Neodiprion lecontei* XP_015522184, Hymenoptera, Orussoidea: *Orussus abietinus* XP_012277050; Coleoptera: *Agrilus planipennis* XP_025834993, *Nicrophorus vespilloides* XP_017778298, *Tribolium castaneum* XP_008190364; Raphidioptera: *Inocellia crassicornis* GAZH02002684, *Xanthostigma xanthostigma* GAUI02021553 (TSA, partial).

*Drosophila melanogaster* has, besides the full-size TPPP ortholog, a protein with a truncated p25alpha domain as well, where the last coding exon is missing. It is not the result of an alternative splicing but is coded by another gene. Interestingly, this shorter protein of 117 aa (CG6709) is encoded by a dicistronic gene (The other protein coded by this open reading frame is CG14164). All the *Drosophila* species, whose genomes have been sequenced, possess an ortholog with the same length. However, in the other species, the CG6709 orthologs are coded by a “normal” gene. Figure 1 shows the sequence alignment of the two *D. melanogaster* TPPP proteins with the human TPPP1. The p25alpha domain starts with a very conserved LxxxFxxF(Y) motif (See it also in Figure 2). It can be seen that the first two exons of the human protein are merged in the long-type CG45057 fruit fly protein but not in the truncated one (CG6709), while the first exon of the human protein is split in the truncated CG6709 protein. Moreover, at the border of the first and second exon (of the human protein), a conserved lysine is encoded by two nucleotides of the first and one nucleotide of the second exon in both the human and the truncated *Drosophila* protein (phase 2 introns). The last exon starts in the same position in the long-type CG45057 protein and in the human one but is missing in the truncated CG6709 protein. The 117 aa long CG6709 orthologs are more similar to each other than to the corresponding long-type TPPPs in the same species and *vice versa*. Based on the comparison of both orthologs in twelve *Drosphila* species, the identity of CG6709 proteins and CG45057 proteins varies between 65.81% and 99.15% and between 79.37% and 100%, respectively (Supplementary Figure 3). However, the pairwise identity of the long-type (186-194 aa) and “truncated” (117 aa) proteins in the same species is between 21.62%–27.27% (average 24.81%).

Blast [8] searches revealed that the orthologs of CG6709 protein can be found not only in all the *Drosophila* species but in both suborders of Diptera. The length of the proteins is 116–119 amino acid (aa) and 111-115 aa in Brachycera and Nematocera, respectively (Figure 2). The first exon is split only in the *Drosophila* species and the very closely related Drosophilini, *Zaprionus indianus*; thus the other orthologs contain only two exons. Orthologs can also be found in other orders of Endopterygota, namely, in Lepidoptera (butterflies and moths; 123-127 aa), Coleoptera (beetles; 116-122 aa) and Hymenoptera (sawflies, wasps, bees, and ants; 106-128 aa) (Figure 2)(It should be noted that databases name these proteins often erroneously as “CG45057-like”. The correct name ought to be CG6709-like). In three Raphidioptera (snakeflies) species (*Fibla maclachlani*, *Inocellia crassicornis*, *Xanthostigma xanthostigma*) hits were identified as nucleotides in the TSA (Transcriptome Shotgun Assembly) database (A detailed list of the CG6709 orthologs are shown in Supplementary Figure 4). Search in the other Endopterygota orders has not resulted in hits yet; however, it should be noted that far less species have been sequenced from these orders than from the above mentioned ones. E.g., search in the WGS (Whole Genome Shotgun) database suggests that Trrichoptera species also contain this gene and after the annotation of these genomes we can receive an unambiguous answer. I was wondering how strong the correlation is between metamorphosis and the presence of CG6709-like gene/protein. For this purpose, the Endopterygota genome assemblies of the https://www.ncbi.nlm.nih.gov/genome webpage, which lists the *completed* projects, were analyzed (Table 1). I found that 85% of the fully sequenced Endopterygota genomes contain CG6709 gene/protein. Orthologs were not found in other insects or in arthropods, although the long-type TPPPs (CG45057 orthologs) are common in these taxonomic units. Thus it seems that this protein occurs only in insects that undergo metamorphosis. However, they are absent in any other organisms.

**Table 1:** Occurrence of CG6709-like gene/protein in Endopterygota.

	Order	Family	Genus	Species
Diptera	1/1	12/15	25/30	85/90
Coleoptera	1/1	6/10	9/19	9/20
Hymenoptera	1/1	15/16	39/56	54/75
Lepidoptera	1/1	19/20	64/65	85/86
Siphonaptera	0/1	0/1	0/1	0/1
**Endopterygota**	4/5	52/62	127/171	233/272
	80.0%	83.9%	74.3%	85.7%

The 554 Endopterygota genome assemblies of the https://www.ncbi.nlm.nih.gov/genome webpage, which lists the *completed* projects (date: 28-02-2021), were analyzed; only the genomes where the protein count was given (272) were used for analysis. Numbers of genomes containing CG6709 versus all genomes are given.

**Figure 3:**
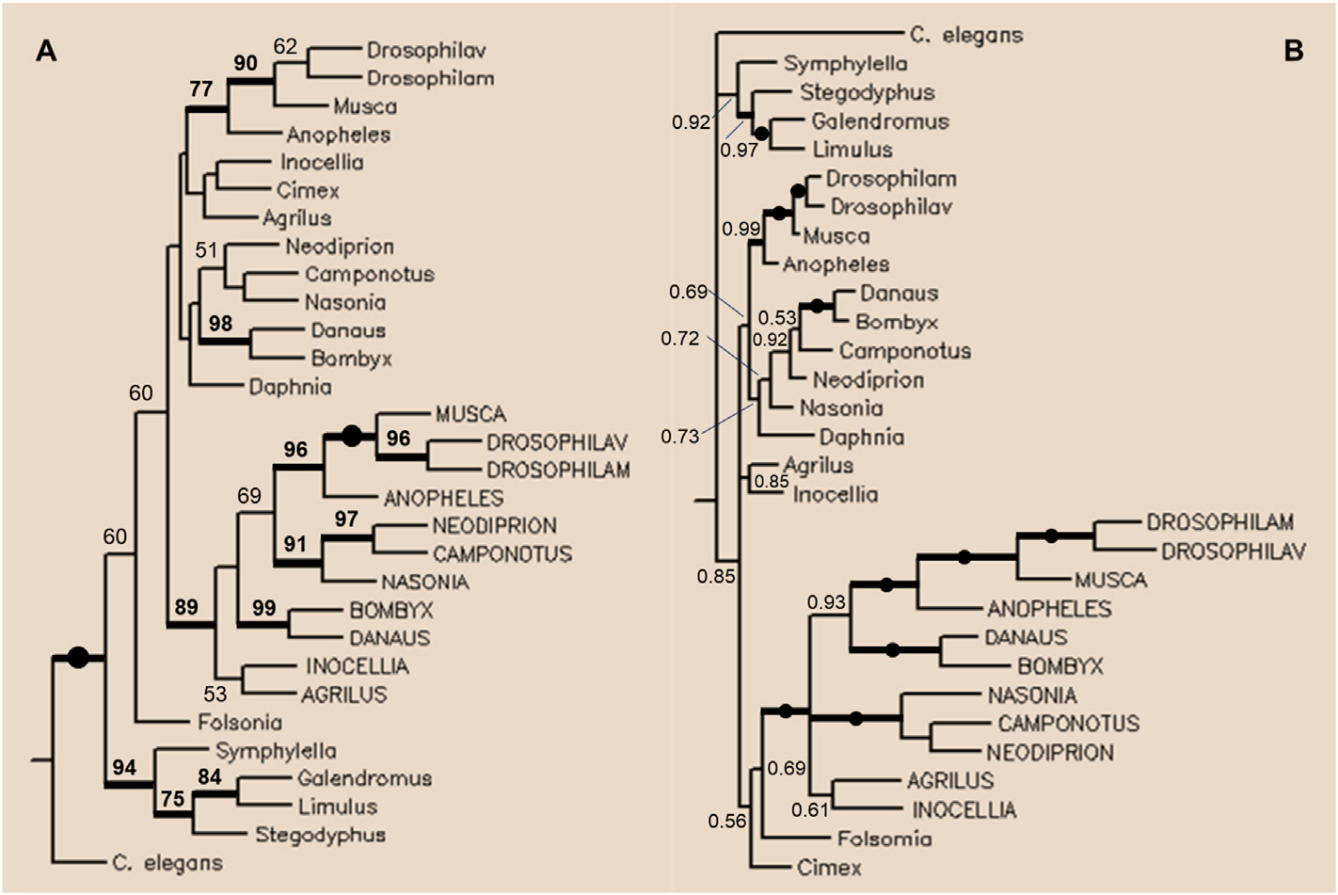
Phylogenetic tree of long and truncated TPPPs of Arthropoda obtained by Maximum Parsimony (A) and Bayesian (B) analysis. (A) Numbers above internal branches indicate bootstrap values shown as percentages (A) and Bayesian posterior probabilities (BPP) (B). Branches that received maximum support are indicated by full circles. Branches with bootstrap support higher than 70% or 0.95 BPP are indicated by thickened lines. Values lower than 50% are not indicated. For easier comparison, truncated TPPPs are labeled by capital letters. Proteins (TSAs∗) used for the construction of the tree: Hexapoda, Insecta, Endopterygota: *Drosophila melanogaster* NP_001246792, NP_648370; *Drosophila virilis* XP_015031007, XP_002047114; *Musca domestica* XP_005178632, XP_005179904; *Anopheles gambiae* XP_308808, XP_556944; *Danaus plexippus* XP_032520671, XP_032527880; *Bombyx mori* XP_004931507, XP_004933177; *Nasonia vitripennis* XP_001604263, XP_008211062; *Camponotus floridanus* XP_011250777. XP_011254991; *Neodiprion lecontei* XP_015521829, XP_015522184; *Agrilus planipennis* XP_018319221; XP_025834993; *Inocellia crassicornis* GAZH02007654∗; GAZH02002684∗; Hexapoda, Insecta, Paraneoptera: *Cimex lectularius* XP_014248365; Hexapoda; Collembola: *Folsomia candida* OXA61843; Crustacea: *Daphnia pulex* EFX79744; Myriopoda: *Symphylella vulgaris* GAKX01025293∗; Chelicerata: *Galendromus (Metaseiulus) occidentalis* XP_003743482; *Limulus polyphemus* XP_013794809; *Stegodyphus mimosarum* KFM57015; Nematoda: *Caenorhabditis elegans* NP_491219 (outgroup).

The phylogenetic analysis included long-type arthropod TPPPs as well as truncated TPPPs found in Endopterygota. Maximum parsimony analysis shows that truncated TPPPs are separated from the full-length (long-type) TPPPs and they are sister groups of each other's (Figure 3A). The N- and C-terminal parts of the long-type proteins were omitted from the alignment used for the construction of the tree since they are absent from the “truncated” ones; i.e., their p25alpha domain was used without the last exon (cf. Figure 1). Thus the tree is based on the common part of the proteins. Bayesian phylogenetic analysis using the program MrBayes v3.1.2 [15] also shows that truncated and long-type TPPPs form a separate clade (Figure 3B). It indicates that the presence of two kinds of TPPPs in Endopterygota species is not the result of in-species/family/order gene duplications but the consequence of an event occurring earlier, in their common Endopterygota ancestor. This is the reason for what can be seen in Supplementary Figure 3: there is a greater evolutionary distance between long-type – truncated TPPP pairs in the *same Drosophila* species than between truncated TPPPs in *different Drosophila* species.

**Figure 4:**
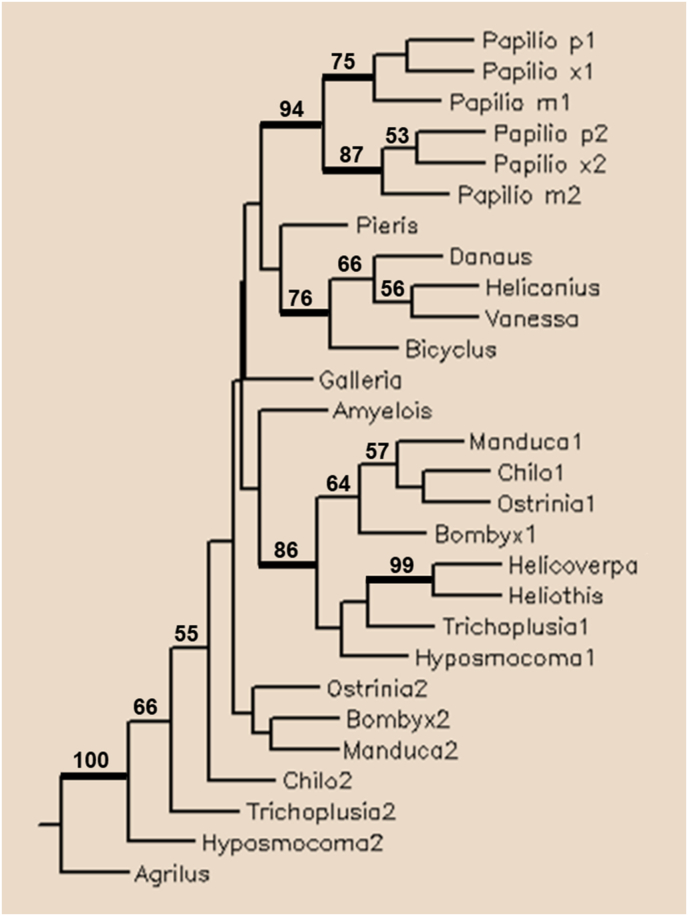
Phylogenetic tree of truncated TPPPs of Lepidoptera obtained by Maximum Parsimony analysis. Numbers above internal branches indicate bootstrap values. Only values higher than 50% are indicated. Branches with bootstrap support higher than 70% are indicated by thickened lines. Proteins (TSAs∗) used for the construction of the tree: Gelechioidea, Cosmopterigidae: *Hyposmocoma kahamanoa* XP_026315514, XP_026315526; Bombycoidea, Bombycidae: *Bombyx mori* XP_004933177, FS874530∗; Bombycoidea, Sphingidae: *Manduca sexta* XP_030040282, XP_030040281; Noctuoidea, Noctuidae: *Trichoplusia ni* XP_026742116, XP_026742114; *Heliothis virescens* PCG65904; *Helicoverpa armigera* XP_021192998; Papilionoidea, Nymphalidae: *Bicyclus anynana* XP_023933890; *Danaus plexippus* XP_032527880; *Heliconius melpomene* HMEL012067 Ensemble.; *Vanessa tameamea* XP_026490343; Papilionoidea, Papilionidae: *Papilio machaon* KPJ10293, KPJ10294; *Papilio polytes* XP_013136554, XP_013136556; *Papilio xuthus* XP_013162360, XP_013162358; Papilionoidea, Pieridae: *Pieris rapae* XP_022125790; Pyraloidea, Pyralidae: *Amyelois transitella* XP_013189465; *Chilo suppressalis* RVE51050, RVE42175; *Galleria mellonella* XP_026762893; *Ostrinia furnacalis* XP_028172140, XP_028169773; Coleoptera: *Agrilus planipennis* XP_025834993 (outgroup).

In Lepidoptera, in several superfamilies (Gelechioidea, Bombycoidea, Noctuoidea, Pyraloidea) two paralogs of the truncated TPPP occur. According to the phylogenetic tree, it seems to be the consequence of an early gene duplication within the order since the two paralogs form separate clades (Figure 4). They can be considered as outparalogs [7] since the duplication event happened earlier than the species speciation. Interestingly, in butterflies (Papilionoidea superfamily), only one truncated TPPP can be found except in *Papilio* species. However, the duplicated presence of the gene/protein in this genus seems to be consequence of a genus (family)-specific gene duplication (Figure 4); i.e., they are inparalogs [7].

## 4 Discussion

The function of this truncated TPPP protein is not known. In the case of other members of the TPPP-like proteins, their binding to the microtubules and their role in stabilizing/organizing cytoskeletal structures were shown. Long-type TPPPs bind tubulin and promote its polymerization into microtubules; and bundle microtubules [2, 6]. This function is conserved in animals [4], including TPPP of *D. melanogaster*, CG45057. The fruit fly protein regulates microtubule stabilization and axonal extension during embryonic development [5], as well as synaptic microtubule organization via the acetylation level of the microtubule network [16]; and acts probably as a hub for microtubule regulators [17].

The amino acid sequences needed for tubulin/microtubule binding are located in the C-terminus of long-type TPPPs [4, 18, 19, 20]. In human TPPP1, there is an additional binding site at the N-terminus; the central part lacks any microtubule binding properties [18, 19, 20]. The insect-specific shorter protein contains practically only the “core” part but not the N- and *C termini* of the long-type TPPP (cf. Figure 1). The amino acid sequences needed for tubulin/microtubule binding located in the C-terminus of long-type TPPPs can be found in another TPPP-like protein, apicortin, occurring mostly in apicomplexan parasites [21]. Indeed, the necessity of this protein for the formation of the structure of the conoid, the nontubular polymeric form of tubulin, was proven [22]. Since the “truncated” TPPP lacks these amino acids thus it is a logical conclusion that it very probably is not able to bind microtubules.

One can speculate, on the basis of its specific phylogenetic occurrence, i.e., that it is present only in Endopterygota, and, otherwise, practically all Endopterygota orders seem to contain this gene, that its function may be related somehow to metamorphosis. According to the gene expression data of the Bgee database (https://bgee.org/; [23]), CG6709 is most abundantly expressed in testis, in pupa and in imaginal discs. During the pupal stage, many larval structures are broken down, and adult structures, including the discs, undergo rapid development [24]. Its abundance in the pupa in general and in the imaginal discs corroborates its potential role during metamorphosis. However, since about 15% of Endopterygota species seem to lack the CG6709 gene/protein, it cannot be excluded that it may have another role. It would mean that Endopterygota species might find a new, yet unknown, function for an old sequence. Obviously, experimental verification of this hypothesis is necessary.

## Declarations

### Author contribution statement

Ferenc Orosz: Conceived and designed the experiments; Performed the experiments; Analyzed and interpreted the data; Contributed reagents, materials, analysis tools or data; Wrote the paper.

### Funding statement

This research did not receive any specific grant from funding agencies in the public, commercial, or not-for-profit sectors.

### Data availability statement

Data included in article/supplementary material/referenced in article.

### Declaration of interests statement

The authors declare no conflict of interest.

### Additional information

No additional information is available for this paper.

## Appendix A Supplementary data

FileS1.docx **File 1:** Multiple sequence alignments of TPPP proteins by Clustal Omega [10] used for constructing the phylogenetic trees in Figure 3. FileS1.docx

FileS2.docx **File 2:** Multiple sequence alignments of TPPP proteins by Clustal Omega [10] used for constructing the phylogenetic tree in Figure 4. FileS2.docx

FileS3.docx **File 3:** The percent identity matrix of CG6709 proteins and CG45057 proteins from twelve *Drosophila* species obtained by Clustal Omega program [10]. FileS3.docx

FileS4.docx **File 4:** List of CG6709 orthologs. FileS4.docx
