# Supplementary material for: Truncated TPPP – An Endopterygota-specific protein
Source: Heliyon. 2021 May 24;7(5):e07135. doi: 10.1016/j.heliyon.2021.e07135 (PMC8180608; doi:10.1016/j.heliyon.2021.e07135)
Supplement: FileS1.docx — File 1: Multiple sequence alignments of TPPP proteins by Clustal Omega [10] used for constructing the phylogenetic trees in Figure 3. [file mmc1.docx]

#NEXUS

[TITLE: Written by EMBOSS 24/03/20]

begin data;

dimensions ntax=30 nchar=114;

format interleave datatype=protein missing=X gap=-;

matrix

Ce VKKRWDAFTKFGAA-------TATEMTGKNFDKWLKDAGVLDNKAITGTM

Dm FSDQFKAFSKFGD-----SKSDGKLITLSQSDKWMKQAKVID-KKITTTD

Dm2 ldslflvysnfqviptdieneyfdsillsqldawleqaklmp-npitrtq

Dv FSDQFKAFSKFGD-----TKSDGKLITLSQSDKWMKQAKVFD-KQITTTD

Dv2 ldslfhvycnhkvigneleneefhsillsqldnwlqqaklmp-vpitrtq

Musca FQDQFKAFSKFGD-----TKSDGKLITLSQSDKWMKQAKVID-KKITTTD

Musca2 leslfhlyannivvtmdldnedydcillsqidywleqakllr-ttftvte

Anopheles FKEQFKAFSKFGD-----TKSDGKHLTLSQSDKWMKQAKVID-KKITTTD

Anopheles2 lpsmftlfakyrptl-nsfqgdgkrillsqsdcwmqqanligpkhftltq

Danaus FKDAFKMFSKFGD-----PKSDGKQITLSQSDKWMKQAKVIDGKKITTTD

Danaus2 ldgqfyefakmmd-----kkrdgttitlynsdfwfrqckilddrkvtmtd

Bombyx FKEAFKAFSKFGD-----PKSDGKAITLSQSDKWMKQAKVIDGKKITTTD

Bombyx2 legqfhefsrlld-----nkrdgntmtlyrsdywmrqskvlddrkvtmtd

Nasonia FIASFKAFSKFGD-----TKSDGKHITLSQSDKWMKQAKVIDGKKITTTD

Nasonia2 lnemfkvycsfdai---sremnvellplsqidkwlyiaqildlhnvtttd

Camponotus FLANFKAFSKFGD-----PKSDGKLITLSQSDKWMKQAKVIDGKKITTTD

Camponotus2 leemfaaycsmdpv---sqrmgvsliplsqsnkwlmsagildmikltttd

Neodiprion FTASFKAFSKFGD-----PKSDGKQITLSQSDKWMKQAKVIDGKKITTTD

Neodiprion2 ldvmfaaycrmdpl---snstgvkllplsqsdkwlasakildmhkvtttd

Agrilus FKEVFRAFSKFGD-----TKSDGRLITLSQSDKWMKQAKVIDGKKITTTD

Agrilus2 mesqfynfaryge-----etadgktitltksdkwmkqakildgknvtltd

Inocellia FKEQFKAFSKFGD-----TKSDGKLITLSQSDKWMKQAKVIDGKKITTTD

Inocellia2 lkeqfvlyskfgd-----ptadgstimlsqadrwlkqaeiidnkkisltd

Cimex YAETFKLFSKFGD-----TKSDGKHITLSQSDKWMKQAKVIDGKTITSTD

Folsomia LNDQFAIFSRFGD-----TASDGKHITLSNSDKWMKQAKVVDGKKITTTD

Daphnia LTELFRAFAKFGD-----SKADGKAISLSQSDKWMKQAKVIDGKKITATD

Symphylella LEEKFRAFAKFGD-----SKSTGDGITLSNSDKWMKQAKVIDGKKITTVD

Stegodyphus FEDQFKLFAKFGD-----SKSTGEAITLSNSDKWMKQAKVIDK-KLTTTD

Galendromus FQEQFKSFAKFGD-----SKSTGDAITLSNSDKWFKQSKVIDGKKITTTD

Limulus FEEMFKAFAKFGD-----TKNTGDSITLFNSDKWFKQSKVIDGKKITTTD

Ce TGIAFSKVTGPKKKATFDETKKVLAFVAEDRARQSKKPIQDELDAITEKL

Dm TGIHFKKFK--AMKISLSDYNKFLDDLAKTKK--------VELSEIKQKL

Dm2 tgliymryk--kwrleyedflevlnnlasdnn--------laidemkqim

Dv TGIHFKKFK--AMKITLADYNKFLEDLAKTKK--------VELAQIKQKM

Dv2 tgllymryk--kwrldyedflevlqhlstdad--------lnyedfkvtl

Musca TGIHFKKFK--AMKISYGDYNKFLEDLAKTKK--------VELQEIKNKM

Musca2 tglvymefr--kwrldyeefldflekicegkn--------vtveevktfl

Anopheles TGIHFKKLK--SMKLTYEDYNKFLDDLAKTKK--------VELDEIKNKM

Anopheles2 tgliffefr--kstldydeylqflallcnekq--------vsveevkekl

Danaus TAIHFKKLK--SVKLGIDDYQKFLEDLAKNKK--------MEVEEIKRKL

Danaus2 tgilfnkfg--kseinwdewneflvdlcelkg--------ldlekaqdtl

Bombyx TAIHFKKLK--SVKLGIDDYQKFLDDLAKNKK--------VELDEIKKKL

Bombyx2 tgvlwwkyc--ktelnwqewydfftdlcelkg--------ldqefvetmm

Nasonia TGIYFKKQK--SMKLSLEQYKAFLGELAKSKK--------VELAEIKSKM

Nasonia2 tglcffkfr--krainyeefleyirdlanikk--------lklddiehkl

Camponotus TGIYFKKHK--STKLGIEQYKTFLDELAKNKK--------VELTEIKKKM

Camponotus2 tglaffkfr--kralsyveyltylkdlatsyn--------lnfedmkyrm

Neodiprion TGIYFKKHK--SVKLGLEQYKAYLEDLAKNKK--------VDVEELKNKM

Neodiprion2 tglcffkfh--kraisfeeyliyledlaqtkg--------lniedmkfam

Agrilus TGIYFKKLK--QLKVPFSDYSKFLEDLAKSKK--------VDLEEMKHKM

Agrilus2 tgfcfskfk--ayaikfddymifledlaqykq--------ldaeeiknkl

Inocellia TGIYFKKLK--SLKVNSEDYKKFLNDLAGSKK--------INVEDIKTKM

Inocellia2 tglcfskfk--akaisfekfitfledfaqyke--------ldlqdikekl

Cimex TGIYFKKLK--QHKVTFADYNKFLEDLAKAKK--------VDVEEIKRKL

Folsomia TSICFKKLFKTTKKVPIEDFKKYIEELAKSKK--------IEPQELLDKL

Daphnia TGIYFKKHK--SLKLGLADYQKFLEELAKAKK--------VELTEIREKM

Symphylella TGIYFKQVAKTKKAVAYKEYKQYLENLCKNKK--------VDFGEISGKM

Stegodyphus TGIYYKMVAKTKRSLSIKEYEQFLETLAKNKK--------TDVSEMKQKM

Galendromus TGIYFKKIAKTKKALTQKEYEQFLDEIAKNKK--------VPLEEIKQKL

Limulus TGIYFKQISKVKKALNLNEYNQFLENIAKNKK--------MDVTEIKQKL

Ce AKLEAPSVGGAA--

Dm ASCGAPGVVSVS--

Dm2 idagvpngadvviv

Dv AGCGAPGVQQVS--

Dv2 vaagpptgateivv

Musca ASCGAPGVIQVS--

Musca2 leagvpgtgssdvv

Anopheles ANCGAPGVHNAT--

Anopheles2 tncgppgits----

Danaus TTCGQPGVSTHLPK

Danaus2 tncglpgqtpvvvp

Bombyx TTCGQPGITSHVTK

Bombyx2 tncgipgsspvlip

Nasonia ANCGPPGVSGAPV-

Nasonia2 rtcalpaekqk---

Camponotus ANCGSPGVTTGSNS

Camponotus2 qicgkpsimredik

Neodiprion ANCGAPGVSSHA--

Neodiprion2 qrcgkpvhpldikk

Agrilus ANCGQPGFSGVG--

Agrilus2 vlcglpgasekqdd

Inocellia TNCGPPECHGTGG-

Inocellia2 idcgppvapkqpkk

Cimex SNCGPPGLSSNVG-

Folsomia TNCGSPSLSTAT--

Daphnia IQCGPPGTTGTT--

Symphylella VNCGQPGLSGTT--

Stegodyphus ASCGPPATSKTT--

Galendromus CACGPPATRIQSA-

Limulus SSCGPPGTSSKTT-

;

end;

begin assumptions;

options deftype=unord;

end;
