# Supplementary material for: Truncated TPPP – An Endopterygota-specific protein
Source: Heliyon. 2021 May 24;7(5):e07135. doi: 10.1016/j.heliyon.2021.e07135 (PMC8180608; doi:10.1016/j.heliyon.2021.e07135)
Supplement: FileS2.docx — File 2: Multiple sequence alignments of TPPP proteins by Clustal Omega [10] used for constructing the phylogenetic tree in Figure 4. [file mmc2.docx]

15 125

Agrilus npetsgftmesqfynfarygee-tadgktitltksdkwmkqakildgknv

PapiliomaxMGEEEAATLEGQFYEFSRLFDN-KRDGTTITLYRFDYWLRQTKLLDDRKI

Papiliomaymgeeeaatlegqfyefsklfdn-krngttitlyrfdywlrqtkilddrnv

PapilioxuxMGEEEAATLEGQFYEFSRLFDN-KRDGTTITLYRFDYWLRQTKLLDDRKV

Papilioxuymgeeeaatlegqfyefsklldn-krdgttitlyrfdywlrqtkilddrkv

PapiliopoxMGDEEAATLEGQFYEFALLFDH-KRNGLTITLYRFDYWLRQTKLLDDRRI

Papiliopoymgeeevatlegqfyefsrlfdn-krdgltitlyrfdywlrqtkllddrkv

Danaus mgeeepasldgqfyefakmmdk-krdgttitlynsdfwfrqckilddrkv

Bombyxx -MEEEEATLEGQFHEFSRLLDN-KRDGNTMTLYRSDYWMRQSKVLDDRKV

Amyelois MGDEEQATLDGQFVDFAKMLDPKGRNGTTINLYRLDYWLRQGKIIDDRKI

Bicyclus MGEEEPATLDGQFYEFGKMMDK-KRDGTTITLYFSDYWFRQAKLLDDRKL

Pieris mgdeeaatlegqfyefsrlldn-krdgttitlyrsdfwmrqakllddrkl

Helicoverpmeeeepatltdqfdkfaklfdn-drdgttidlyrsdywlrqsnvledral

Heliothis -meeepatltdqlvafaklfdi-drdgttidlyrsdywlrqskvledrel

HeliconiusMGEEEPASLEGQFFEYAKMMDK-KRDGTTITLYNSDYWMRQCKLLDDRKL

tltdtgfcfskfkayaikfddymifledlaqykqldaeeiknklvlcglp

TMTDTGILFNKFNKTELNWDEWMEFLEDLCELKKMDLEKTKETLTNCGLP

tmtdtgisfnkfdktelnwdeflefledlcelkkmdfektketltncglp

TMTDTGILFNKFNKTELNWDEWMEFLEDLCELKEMDLEKTKETLTNCGLP

tmtdtgiafnkfnktelnwdeflefledlcelkdmdlektketltncglp

TMTDTGISFNKFNKAELNWDEFLEFLEDLCEMKDMDLEKMKETLTNCGLP

tmtdtgilfnkynktelnwdewsefledlcelkemdlektketltncglp

tmtdtgilfnkfgkseinwdewneflvdlcelkgldlekaqdtltncglp

TMTDTGVLWWKYCKTELNWQEWYDFFTDLCELKGLDQEFVETMMTNCGIP

TMTDTGIIFNKFFKSEIDYDEFLEFLEDLANLKSLYLEKLQETLTNCGLP

TMTDTGIAFNKFSKTELNWDEWNEYLTDICQTKELDEEKVRETLTNCGLP

tmtdtgilfnkfskteldwdewnqfldevcelkqfdeekvretltncglp

tmtdtgiawmkfskteltydewyqiladfctsrgldrasveaaltncglp

tmtdtgivwmklskieltfsewyemlgeictrrgldrdsveaaltncglp

TMTDTGILFNKFSKSELNWDEWNEFLTELCELKELEEEKVKDLMTNCGLP

gasekqddkkrrr------------

GQTPVNVPQYRDFFLTYKPKEKMAF

gqtpvevpqfrdyfltykpkekaaf

GQTPVNVPQYRDFFLTYKPKEKMIF

gqlpvavpqfrdyfltykpkeksay

GQTPILVPQFRDYFLTYKPKEKSAY

gqtpvnvpqyrdffltykprekmil

gqtpvvvpqyrdffltykpkekmlf

GSSPVLIPQFRDFFDTFKPKEKLPF

GSCAVFVPQYRTFFDTYKPKEKMLF

GQSPVVVPQYRDFFLTYKPKERMLF

gqtpvlvpqyrdffatykpkeklpf

gsvnvnvpqyrdlfdtvkpppklly

gtikvfvpqyrdmyytykprsklin

GQTPVFVPQYRDFFLTYKPKEKMLF
