# Supplementary material for: Truncated TPPP – An Endopterygota-specific protein
Source: Heliyon. 2021 May 24;7(5):e07135. doi: 10.1016/j.heliyon.2021.e07135 (PMC8180608; doi:10.1016/j.heliyon.2021.e07135)
Supplement: FileS3.docx — File 3: The percent identity matrix of CG6709 proteins and CG45057 proteins from twelve Drosophila species obtained by Clustal Omega program [10]. [file mmc3.docx]

The percent identity matrix of CG6709 proteins and CG45057 proteins from twelve *Drosophila* species obtained by Clustal Omega program (Sievers *et al*., 2011).

Percent Identity Matrix - created by Clustal 2.1

1 2 3 4 5 6 7 8 9 10 11 12

13 14 15 16 17 18 19 20 21 22 23 24

1: Dg 100.00 87.63 89.78 79.37 82.35 85.86 85.86 82.45 81.48 82.01 83.07 83.07

**21.62** 27.03 24.32 27.03 29.73 28.83 28.83 27.93 27.93 25.23 25.23 25.23

2: Dmo 87.63 100.00 92.47 83.06 85.08 85.95 85.95 82.42 81.97 83.61 83.06 83.06

20.72 24.32 22.52 25.23 27.93 27.93 27.93 27.03 27.03 25.23 24.32 24.32

3: Dv 89.78 92.47 100.00 85.25 90.06 88.65 88.65 85.16 85.25 85.79 86.89 85.79

20.72 23.42 21.62 24.32 27.03 27.03 27.03 26.13 26.13 24.32 23.42 23.42

4: Dw 79.37 83.06 85.25 100.00 88.89 85.79 85.79 85.03 84.57 85.64 85.64 85.11

22.52 27.03 24.32 25.23 27.93 27.03 27.03 26.13 26.13 25.23 26.13 26.13

5: Da 82.35 85.08 90.06 88.89 100.00 88.95 88.95 88.77 89.36 89.36 89.89 89.36

20.54 25.89 22.32 25.00 26.79 25.89 25.89 25.00 25.00 26.79 25.89 25.89

6: Dpe 85.86 85.95 88.65 85.79 88.95 100.00 100.00 89.53 89.06 90.10 90.10 89.58

22.32 25.89 24.11 25.00 28.57 27.68 27.68 26.79 26.79 26.79 25.89 25.89

7: Dps 85.86 85.95 88.65 85.79 88.95 100.00 100.00 89.53 89.06 90.10 90.10 89.58

22.32 25.89 24.11 25.00 28.57 27.68 27.68 26.79 26.79 26.79 25.89 25.89

8: Dsi 82.45 82.42 85.16 85.03 88.77 89.53 89.53 100.00 97.38 98.43 98.43 98.95

20.91 26.36 22.73 25.45 27.27 26.36 26.36 25.45 25.45 27.27 26.36 26.36

9: Dy 81.48 81.97 85.25 84.57 89.36 89.06 89.06 97.38 100.00 97.92 98.44 97.40

20.91 26.36 22.73 25.45 27.27 26.36 26.36 25.45 25.45 27.27 26.36 26.36

10: Dm 82.01 83.61 85.79 85.64 89.36 90.10 90.10 98.43 97.92 100.00 98.96 98.44

20.91 26.36 22.73 25.45 **27.27** 26.36 26.36 25.45 25.45 27.27 26.36 26.36

11: De 83.07 83.06 86.89 85.64 89.89 90.10 90.10 98.43 98.44 98.96 100.00 98.44

20.91 26.36 22.73 25.45 27.27 26.36 26.36 25.45 25.45 27.27 26.36 26.36

12: Dse 83.07 83.06 85.79 85.11 89.36 89.58 89.58 98.95 97.40 98.44 98.44 100.00

20.91 26.36 22.73 25.45 27.27 26.36 26.36 25.45 25.45 27.27 26.36 26.36

13: Dg2 21.62 20.72 20.72 22.52 20.54 22.32 22.32 20.91 20.91 20.91 20.91 20.91

100.00 77.78 76.92 68.38 67.52 67.52 67.52 68.38 65.81 73.50 70.09 69.23

14: Dmo2 27.03 24.32 23.42 27.03 25.89 25.89 25.89 26.36 26.36 26.36 26.36 26.36

77.78 100.00 82.05 69.23 70.09 69.23 69.23 68.38 70.94 72.65 70.94 71.79

15: Dv2 24.32 22.52 21.62 24.32 22.32 24.11 24.11 22.73 22.73 22.73 22.73 22.73

76.92 82.05 100.00 70.94 68.38 69.23 69.23 68.38 70.94 73.50 71.79 72.65

16: Da2 27.03 25.23 24.32 25.23 25.00 25.00 25.00 25.45 25.45 25.45 25.45 25.45

68.38 69.23 70.94 100.00 78.63 78.63 78.63 80.34 76.92 77.78 76.92 76.07

17: Dm2 29.73 27.93 27.03 27.93 26.79 28.57 28.57 27.27 27.27 27.27 27.27 27.27

67.52 70.09 68.38 78.63 100.00 98.29 98.29 94.02 93.16 73.50 70.94 70.09

18: Dse2 28.83 27.93 27.03 27.03 25.89 27.68 27.68 26.36 26.36 26.36 26.36 26.36

67.52 69.23 69.23 78.63 98.29 100.00 100.00 94.87 94.02 72.65 70.09 69.23

19: Dsi2 28.83 27.93 27.03 27.03 25.89 27.68 27.68 26.36 26.36 26.36 26.36 26.36

67.52 69.23 69.23 78.63 98.29 100.00 100.00 94.87 94.02 72.65 70.09 69.23

20: De2 27.93 27.03 26.13 26.13 25.00 26.79 26.79 25.45 25.45 25.45 25.45 25.45

68.38 68.38 68.38 80.34 94.02 94.87 94.87 100.00 95.73 72.65 70.94 70.09

21: Dy2 27.93 27.03 26.13 26.13 25.00 26.79 26.79 25.45 25.45 25.45 25.45 25.45

65.81 70.94 70.94 76.92 93.16 94.02 94.02 95.73 100.00 70.94 69.23 70.09

22: Dw2 25.23 25.23 24.32 25.23 26.79 26.79 26.79 27.27 27.27 27.27 27.27 27.27

73.50 72.65 73.50 77.78 73.50 72.65 72.65 72.65 70.94 100.00 78.63 77.78

23: Dpe2 25.23 24.32 23.42 26.13 25.89 25.89 25.89 26.36 26.36 26.36 26.36 26.36

70.09 70.94 71.79 76.92 70.94 70.09 70.09 70.94 69.23 78.63 100.00 99.15

24: Dps2 25.23 24.32 23.42 26.13 25.89 25.89 25.89 26.36 26.36 26.36 26.36 26.36

69.23 71.79 72.65 76.07 70.09 69.23 69.23 70.09 70.09 77.78 99.15 100.00

Numbers 1-12: CG45057 (long) *Drosophila* TPPP proteins, numbers 13-14: CG6709 (“truncated”) *Drosophila* TPPP proteins. Dark backgrounds label the pairwise identities in the same species (i.e., the identity between the CG45057 and CG6709 proteins of a given species). Da, [*D. ananassae*](http://www.uniprot.org/taxonomy/7217); De, [*D. erecta*](http://www.uniprot.org/taxonomy/7220); Dg, [*D. grimshawi*](http://www.uniprot.org/taxonomy/7222)*,* Dm, [*D. melanogaster*](http://www.uniprot.org/taxonomy/7227); Dmo, [*D. mojavensis*](http://www.uniprot.org/taxonomy/7230); Dpe, [*D. persimilis*](http://www.uniprot.org/taxonomy/7234); Dps, [*D. pseudoobscura*](http://www.uniprot.org/taxonomy/7237); Dse*,* [*D. sechellia*](http://www.uniprot.org/taxonomy/7238); Dsi, [*D. simulans*](http://www.uniprot.org/taxonomy/7240); Dv, [*D. virilis*](http://www.uniprot.org/taxonomy/7244); Dw, [*D. willistoni*](http://www.uniprot.org/taxonomy/7260); Dy, [*D. yakuba*](http://www.uniprot.org/taxonomy/7245)*.*
