# Supplementary material for: Truncated TPPP – An Endopterygota-specific protein
Source: Heliyon. 2021 May 24;7(5):e07135. doi: 10.1016/j.heliyon.2021.e07135 (PMC8180608; doi:10.1016/j.heliyon.2021.e07135)
Supplement: FileS4.docx — File 4: List of CG6709 orthologs. [file mmc4.docx]

**Truncated TPPP (CG6709) orthologs**

| Order | Suborder | Family | Species | Accession number | Length (aa) |
| --- | --- | --- | --- | --- | --- |
| Diptera | Brachycera | Calliphoridae | *Lucilia cuprina* | KNC33086 | 118 |
|  |  |  | *Phormia regina* | MINJ01166604^c^ |  |
|  |  | Diopsoidea | *Sphyracephala brevicornis* | JXPL01003860^c^ | 117 |
|  |  |  | *Teleopsis dalmanni* | XP_037940036 | 117 |
|  |  |  | *Teleopsis whitei* | GBBQ01019489^b^ | 117 |
|  |  | Drosophilidae | [*Drosophila*^f^ *albomicans*](https://www.ncbi.nlm.nih.gov/Taxonomy/Browser/wwwtax.cgi?id=7291) | [XP_034106340](https://www.ncbi.nlm.nih.gov/protein/XP_034106340.1?report=genbank&log$=prottop&blast_rank=29&RID=08MCBZ8W016) | 118 |
|  |  |  | *Drosophila melanogaster* | NP_001097567 | 117 |
|  |  |  | *Phortica variegata* | JXPM01004483^c^ | 117 |
|  |  |  | *Scaptodrosophila lebanonensis* | XP_030385584 | 116 |
|  |  |  | *Zaprionus indianus* | LWKS01010274^c^ | 116 |
|  |  | Ephydridae | *Ephydra gracilis* | JXPQ010028e^c^ | 116 |
|  |  | Glossinidae | *Glossina fuscipes* | XP_037880913 | 117 |
|  |  | Muscidae | *Musca domestica* | XP_005179904 | 118 |
|  |  |  | *Stomoxys calcitrans* | XP_013115971 | 119 |
|  |  | [Sarcophagidae](https://www.ncbi.nlm.nih.gov/Taxonomy/Browser/wwwtax.cgi?mode=Undef&id=7381&lvl=3&lin=f&keep=1&srchmode=1&unlock) | *Neobellieria bullata* | JXPI0108632^c^ | 118 |
|  |  | [Phoridae](https://www.ncbi.nlm.nih.gov/Taxonomy/Browser/wwwtax.cgi?mode=Undef&id=36164&lvl=3&lin=f&keep=1&srchmode=1&unlock) | *Megaselia abdita* | JXPG01069749^c^ | 117 |
|  |  | [Syrphidae](https://www.ncbi.nlm.nih.gov/Taxonomy/Browser/wwwtax.cgi?mode=Undef&id=34680&lvl=3&lin=f&keep=1&srchmode=1&unlock) | *Eristalis dimidiata* | JXPC01002530^c^ | 117 |
|  |  | Stratiomyidae | *Hermetia illucens* | XP_037906735 | 115 |
|  |  | Tephritidae | *Bactrocera dorsalis* | XP_011210810 | 118 |
|  |  |  | *Bactrocera oleae* | XP_014093326 | 118 |
|  |  |  | *Bactrocera latifrons* | [XP_018788896](https://www.ncbi.nlm.nih.gov/protein/XP_018788896.1?report=genbank&log$=prottop&blast_rank=75&RID=0869BDAH013) | 118 |
|  |  |  | *Ceratitis capitata* | XP_004530835 | 118 |
|  |  |  | *Rhagoletis zephyria* | XP_017478284 | 118 |
|  |  |  | [*Rhagoletis pomonella*](https://www.ncbi.nlm.nih.gov/Taxonomy/Browser/wwwtax.cgi?id=28610) | [XP_036335560](https://www.ncbi.nlm.nih.gov/protein/XP_036335560.1?report=genbank&log$=prottop&blast_rank=14&RID=0869BDAH013) | 118 |
|  |  |  | *Zeugodacus cucurbitae* | XP_011183601 | 118 |
|  | Nematocera | [Culicidae](https://www.ncbi.nlm.nih.gov/Taxonomy/Browser/wwwtax.cgi?mode=Undef&id=7157&lvl=3&lin=f&keep=1&srchmode=1&unlock) | *Aedes aegypti* | XP_021695436 | 112 |
|  |  |  | *Aedes albopictus* | XP_019531996 | 112 |
|  |  |  | [*Anopheles*^f^ *albimanus*](https://www.ncbi.nlm.nih.gov/Taxonomy/Browser/wwwtax.cgi?id=7167) | XP_035786950 | 115 |
|  |  |  | *Anopheles darlingi* | ADMH02000262^c^ | 113 |
|  |  |  | *Anopheles gambiae* | XP_556944 | 115 |
|  |  |  | *Anopheles sinensis* | KFB36011 | 112 |
|  |  |  | *Anopheles stephensi* | [XP_035905953](https://www.ncbi.nlm.nih.gov/protein/XP_035905953.1?report=genbank&log$=prottop&blast_rank=5&RID=086SRR7P016) | 111 |
|  |  |  | *Culex quinquefasciatus* | XP_001862283 | 113 |
|  |  |  | *Culex tarsalis* | JAV30645 | 113 |
| Coleoptera | Polyphaga | Buprestidae | *Agrilus planipennis* | XP_025834993 | 122 |
|  |  | Cerambycidae | *Anoplophora glabripennis* | XP_018575094 | 118 |
|  |  | Elateridae | *Ignelater luminosus* | KAF2903478 | 116 |
|  |  | Lampyridae | *Abscondita terminalis* | KAF5305595 | 114 |
|  |  |  | *Lamprigera yunnana* | KAF5303975 | 112 |
|  |  |  | *Photinus pyralis* | XP_031347450 | 110 |
|  |  | Silphidae | *Nicrophorus vespilloides* | XP_017778298 | 119 |
|  |  | Tenebrionidae | *Tribolium castaneum* | XP_008190364 | 117 |
| Hymenoptera | Apocrita | [**Apidae**](https://www.ncbi.nlm.nih.gov/Taxonomy/Browser/wwwtax.cgi?mode=Tree&id=7458&lvl=2&lin=f&keep=1&srchmode=1&unlock) | *Apis dorsata* | XP_006607661 | 113 |
|  |  |  | *Ceratina calcarata* | XP_017881425 | 114 |
|  |  |  | *Eufriesea mexicana* | XP_017754460 | 113 |
|  |  |  | *Habropoda laboriosa* | XP_017791104 | 106 |
|  |  |  | *Melipona quadrifasciata* | KOX72035 | 114 |
|  |  | [Braconidae](https://www.ncbi.nlm.nih.gov/Taxonomy/Browser/wwwtax.cgi?mode=Undef&id=7402&lvl=2&keep=1&srchmode=1&unlock) | *Diachasma alloeum* | XP_015108859 | 124 |
|  |  |  | *Fopius arisanus* | XP_011306785 | 128 |
|  |  |  | *Microplitis demolitor* | XP_008559832 | 116 |
|  |  | Cephidae | *Cephus cinctus* | XP_024943230 | 119 |
|  |  | Cynipidae | *Belonocnema treatae* | XP_033210488 | 117 |
|  |  | [Formicidae](https://www.ncbi.nlm.nih.gov/Taxonomy/Browser/wwwtax.cgi?mode=Undef&id=36668&lvl=2&keep=1&srchmode=1&unlock) | *Camponotus floridanus* | XP_011254991 | 117 |
|  |  |  | *Wasmannia auropunctata* | XP_011705182 | 119 |
|  |  | [**Megachilidae**](https://www.ncbi.nlm.nih.gov/Taxonomy/Browser/wwwtax.cgi?mode=Tree&id=124286&lvl=2&lin=f&keep=1&srchmode=1&unlock) | *Megachile rotundata* | XP_012144914 | 115 |
|  |  | [Pteromalidae](https://www.ncbi.nlm.nih.gov/Taxonomy/Browser/wwwtax.cgi?mode=Undef&id=7423&lvl=2&keep=1&srchmode=1&unlock) | *Nasonia vitripennis* | XP_008211062 | 115 |
|  |  | [Trichogrammatidae](https://www.ncbi.nlm.nih.gov/Taxonomy/Browser/wwwtax.cgi?mode=Undef&id=7489&lvl=2&keep=1&srchmode=1&unlock) | *Trichogramma pretiosum* | XP_014233961 | 128 |
|  |  | [Vespidae](https://www.ncbi.nlm.nih.gov/Taxonomy/Browser/wwwtax.cgi?mode=Undef&id=7438&lvl=2&keep=1&srchmode=1&unlock) | *Polistes dominula* | XP_015188463 | 115 |
|  |  |  | *Vespa mandarinia* | XP_035722278.1 | 115 |
|  | Orussoidea^a^ | [Orussidae](https://www.ncbi.nlm.nih.gov/Taxonomy/Browser/wwwtax.cgi?mode=Undef&id=27528&lvl=2&keep=1&srchmode=1&unlock) | *Orussus abietinus* | XP_012277050 | 127 |
|  | Tenthredinoidea^a^ | [Tenthredinidae](https://www.ncbi.nlm.nih.gov/Taxonomy/Browser/wwwtax.cgi?mode=Undef&id=27532&lvl=2&lin=f&keep=1&srchmode=1&unlock) | *Athalia rosae* | XP_012258014 | 123 |
|  |  |  |  | XP_025602797 | 118 |
|  |  | [Diprionidae](https://www.ncbi.nlm.nih.gov/Taxonomy/Browser/wwwtax.cgi?mode=Undef&id=52632&lvl=2&keep=1&srchmode=1&unlock) | *Neodiprion lecontei* | XP_015522184 | 118 |
| Lepidoptera | Glossata | Bombycidae | *Bombyx mori* | XP_004933177 | 123 |
|  |  |  |  | FS874530^b^ | 124 |
|  |  | Cosmopterigidae | *Hyposmocoma kahamanoa* | XP_026315526  XP_026315514 | 123  124 |
|  |  | Crambidae | *Chilo suppressalis* | RVE51050 | 124 |
|  |  |  |  | RVE42175 | 124 |
|  |  |  | *Ostrinia furnacalis* | XP_028172140 | 123 |
|  |  |  |  | XP_028169773 | 124 |
|  |  | Erebidae | *Arctia plantaginis* | CAB3258974 | 123 |
|  |  | Geometridae | *Operophtera brumata* | KOB73504 | ?^e^ |
|  |  | [Noctuoidae](https://www.ncbi.nlm.nih.gov/Taxonomy/Browser/wwwtax.cgi?mode=Undef&id=37570&lvl=3&lin=f&keep=1&srchmode=1&unlock) | *Helicoverpa armigera* | XP_021192998 | 124 |
|  |  |  | *Heliothis virescens* | PCG65904 | 127 |
|  |  |  | *Spodoptera frugiperda* | XP_035434602 | 131 |
|  |  |  | *Trichoplusia ni* | XP_026742116 | 123 |
|  |  |  |  | XP_026742114 | 124 |
|  |  | [Nymphalidae](https://www.ncbi.nlm.nih.gov/Taxonomy/Browser/wwwtax.cgi?mode=Undef&id=33415&lvl=3&lin=f&keep=1&srchmode=1&unlock) | *Bicyclus anynana* | XP_023933890 | 124 |
|  |  |  | *Danaus plexippus* | XP_032527880 | 124 |
|  |  |  | *Heliconius melpomene* | [HMEL012067](http://metazoa.ensembl.org/Heliconius_melpomene/Gene/Summary?db=core;g=HMEL012067;r=HE671255:80477-81214;t=HMEL012067-RA)^d^ | 124 |
|  |  |  | *Vanessa tameamea* | XP_026490343 | 124 |
|  |  | Papilionidae | *Papilio machaon* | KPJ10293 | 124 |
|  |  |  |  | KPJ10294 | 124 |
|  |  |  | *Papilio polytes* | XP_013136556 | 124 |
|  |  |  |  | XP_013136554 | 124 |
|  |  |  | *Papilio xuthus* | XP_013162360 | 124 |
|  |  |  |  | XP_013162358 | 124 |
|  |  | [Pieridae](https://www.ncbi.nlm.nih.gov/Taxonomy/Browser/wwwtax.cgi?mode=Undef&id=7114&lvl=3&lin=f&keep=1&srchmode=1&unlock) | *Pieris rapae* | XP_022125790 | 124 |
|  |  |  | *Zerene cesonia* | XP_038216668 | 124 |
|  |  | [Pyralidae](https://www.ncbi.nlm.nih.gov/Taxonomy/Browser/wwwtax.cgi?mode=Undef&id=7135&lvl=3&keep=1&srchmode=1&unlock) | *Amyelois transitella* | XP_013189465 | 125 |
|  |  |  | *Galleria mellonella* | XP_026762893 | 124 |
|  |  | Sphingidae | *Manduca sexta* | XP_030040282 | 123 |
|  |  |  |  | XP_030040281 | 124 |
| Raphidioptera |  | [**Inocelliidae**](https://www.ncbi.nlm.nih.gov/Taxonomy/Browser/wwwtax.cgi?mode=Tree&id=140688&lvl=2&lin=f&keep=1&srchmode=1&unlock) | *Inocellia crassicornis* | GAZH02002684^b^ | 120 |
|  |  |  | *Fibla maclachlani* | GCSN01031416 ^b^ | 127 |
|  |  | [**Raphidiidae**](https://www.ncbi.nlm.nih.gov/Taxonomy/Browser/wwwtax.cgi?mode=Tree&id=50483&lvl=2&lin=f&keep=1&srchmode=1&unlock) | *Xanthostigma xanthostigma* | GAUI02021553^b^ | ?^e^ |

Notes: ^a^ – superfamily; ^b^ – TSA (Transcriptome Shotgun Assembly); ^c^ – WGS (whole genome shotgun); ^d^ – Ensemble; ^e^ – incomplete sequence; ^f^ – See detailed list of the genus separately.

***Drosophila* CG6709 orthologs**

| Species | Accession number | Length (aa) |
| --- | --- | --- |
| [*Drosophila melanogaster*](https://www.ncbi.nlm.nih.gov/Taxonomy/Browser/wwwtax.cgi?id=7227) | [NP_001097567](https://www.ncbi.nlm.nih.gov/protein/NP_001097567.1?report=genbank&log$=prottop&blast_rank=1&RID=08MCBZ8W016) | 117 |
| [*Drosophila sechellia*](https://www.ncbi.nlm.nih.gov/Taxonomy/Browser/wwwtax.cgi?id=7238) | [XP_002029959](https://www.ncbi.nlm.nih.gov/protein/XP_002029959.1?report=genbank&log$=prottop&blast_rank=2&RID=08MCBZ8W016) | 117 |
| [*Drosophila mauritiana*](https://www.ncbi.nlm.nih.gov/Taxonomy/Browser/wwwtax.cgi?id=7226) | [XP_033161452](https://www.ncbi.nlm.nih.gov/protein/XP_033161452.1?report=genbank&log$=prottop&blast_rank=3&RID=08MCBZ8W016) | 117 |
| [*Drosophila erecta*](https://www.ncbi.nlm.nih.gov/Taxonomy/Browser/wwwtax.cgi?id=7220) | [XP_001972246](https://www.ncbi.nlm.nih.gov/protein/XP_001972246.1?report=genbank&log$=prottop&blast_rank=4&RID=08MCBZ8W016) | 117 |
| [*Drosophila yakuba*](https://www.ncbi.nlm.nih.gov/Taxonomy/Browser/wwwtax.cgi?id=7245) | [XP_002094265](https://www.ncbi.nlm.nih.gov/protein/XP_002094265.1?report=genbank&log$=prottop&blast_rank=5&RID=08MCBZ8W016) | 117 |
| [*Drosophila rhopaloa*](https://www.ncbi.nlm.nih.gov/Taxonomy/Browser/wwwtax.cgi?id=1041015) | [XP_016971728](https://www.ncbi.nlm.nih.gov/protein/XP_016971728.1?report=genbank&log$=prottop&blast_rank=6&RID=08MCBZ8W016) | 117 |
| [*Drosophila suzukii*](https://www.ncbi.nlm.nih.gov/Taxonomy/Browser/wwwtax.cgi?id=28584) | [XP_016934056](https://www.ncbi.nlm.nih.gov/protein/XP_016934056.1?report=genbank&log$=prottop&blast_rank=7&RID=08MCBZ8W016) | 117 |
| [*Drosophila biarmipes*](https://www.ncbi.nlm.nih.gov/Taxonomy/Browser/wwwtax.cgi?id=125945) | [XP_016965806](https://www.ncbi.nlm.nih.gov/protein/XP_016965806.1?report=genbank&log$=prottop&blast_rank=8&RID=08MCBZ8W016) | 117 |
| [*Drosophila takahashii*](https://www.ncbi.nlm.nih.gov/Taxonomy/Browser/wwwtax.cgi?id=29030) | [XP_016997867](https://www.ncbi.nlm.nih.gov/protein/XP_016997867.1?report=genbank&log$=prottop&blast_rank=9&RID=08MCBZ8W016) | 117 |
| [*Drosophila eugracilis*](https://www.ncbi.nlm.nih.gov/Taxonomy/Browser/wwwtax.cgi?id=29029) | [XP_017080732](https://www.ncbi.nlm.nih.gov/protein/XP_017080732.1?report=genbank&log$=prottop&blast_rank=10&RID=08MCBZ8W016) | 117 |
| [*Drosophila elegans*](https://www.ncbi.nlm.nih.gov/Taxonomy/Browser/wwwtax.cgi?id=30023) | [XP_017133876](https://www.ncbi.nlm.nih.gov/protein/XP_017133876.1?report=genbank&log$=prottop&blast_rank=11&RID=08MCBZ8W016) | 117 |
| [*Drosophila ficusphila*](https://www.ncbi.nlm.nih.gov/Taxonomy/Browser/wwwtax.cgi?id=30025) | [XP_017040392](https://www.ncbi.nlm.nih.gov/protein/XP_017040392.1?report=genbank&log$=prottop&blast_rank=12&RID=08MCBZ8W016) | 117 |
| [*Drosophila serrata*](https://www.ncbi.nlm.nih.gov/Taxonomy/Browser/wwwtax.cgi?id=7274) | [XP_020816893](https://www.ncbi.nlm.nih.gov/protein/XP_020816893.1?report=genbank&log$=prottop&blast_rank=13&RID=08MCBZ8W016) | 117 |
| [*Drosophila ananassae*](https://www.ncbi.nlm.nih.gov/Taxonomy/Browser/wwwtax.cgi?id=7217) | [XP_001957775](https://www.ncbi.nlm.nih.gov/protein/XP_001957775.1?report=genbank&log$=prottop&blast_rank=14&RID=08MCBZ8W016) | 117 |
| [*Drosophila bipectinata*](https://www.ncbi.nlm.nih.gov/Taxonomy/Browser/wwwtax.cgi?id=42026) | [XP_017109011](https://www.ncbi.nlm.nih.gov/protein/XP_017109011.1?report=genbank&log$=prottop&blast_rank=15&RID=08MCBZ8W016) | 117 |
| [*Drosophila kikkawai*](https://www.ncbi.nlm.nih.gov/Taxonomy/Browser/wwwtax.cgi?id=30033) | [XP_017028432](https://www.ncbi.nlm.nih.gov/protein/XP_017028432.1?report=genbank&log$=prottop&blast_rank=16&RID=08MCBZ8W016) | 117 |
| [*Drosophila obscura*](https://www.ncbi.nlm.nih.gov/Taxonomy/Browser/wwwtax.cgi?id=7282) | [XP_022217691](https://www.ncbi.nlm.nih.gov/protein/XP_022217691.1?report=genbank&log$=prottop&blast_rank=17&RID=08MCBZ8W016) | 117 |
| [*Drosophila willistoni*](https://www.ncbi.nlm.nih.gov/Taxonomy/Browser/wwwtax.cgi?id=7260) | [XP_002062203](https://www.ncbi.nlm.nih.gov/protein/XP_002062203.1?report=genbank&log$=prottop&blast_rank=19&RID=08MCBZ8W016) | 117 |
| [*Drosophila subobscura*](https://www.ncbi.nlm.nih.gov/Taxonomy/Browser/wwwtax.cgi?id=7241) | [XP_034658329](https://www.ncbi.nlm.nih.gov/protein/XP_034658329.1?report=genbank&log$=prottop&blast_rank=20&RID=08MCBZ8W016) | 117 |
| [*Drosophila miranda*](https://www.ncbi.nlm.nih.gov/Taxonomy/Browser/wwwtax.cgi?id=7229) | [XP_017136192](https://www.ncbi.nlm.nih.gov/protein/XP_017136192.1?report=genbank&log$=prottop&blast_rank=21&RID=08MCBZ8W016) | 117 |
| [*Drosophila persimilis*](https://www.ncbi.nlm.nih.gov/Taxonomy/Browser/wwwtax.cgi?id=7234) | [XP_002025402](https://www.ncbi.nlm.nih.gov/protein/XP_002025402.1?report=genbank&log$=prottop&blast_rank=22&RID=08MCBZ8W016) | 117 |
| [*Drosophila guanche*](https://www.ncbi.nlm.nih.gov/Taxonomy/Browser/wwwtax.cgi?id=7266) | [XP_034122829](https://www.ncbi.nlm.nih.gov/protein/XP_034122829.1?report=genbank&log$=prottop&blast_rank=23&RID=08MCBZ8W016) | 117 |
| [*Drosophila pseudoobscura*](https://www.ncbi.nlm.nih.gov/Taxonomy/Browser/wwwtax.cgi?id=7237) | [XP_001353716](https://www.ncbi.nlm.nih.gov/protein/XP_001353716.1?report=genbank&log$=prottop&blast_rank=24&RID=08MCBZ8W016) | 117 |
| [*Drosophila busckii*](https://www.ncbi.nlm.nih.gov/Taxonomy/Browser/wwwtax.cgi?id=30019) | [XP_017843842](https://www.ncbi.nlm.nih.gov/protein/XP_017843842.1?report=genbank&log$=prottop&blast_rank=25&RID=08MCBZ8W016) | 117 |
| [*Drosophila innubila*](https://www.ncbi.nlm.nih.gov/Taxonomy/Browser/wwwtax.cgi?id=198719) | [XP_034480314](https://www.ncbi.nlm.nih.gov/protein/XP_034480314.1?report=genbank&log$=prottop&blast_rank=26&RID=08MCBZ8W016) | 117 |
| [*Drosophila mojavensis*](https://www.ncbi.nlm.nih.gov/Taxonomy/Browser/wwwtax.cgi?id=7230) | [XP_002007566](https://www.ncbi.nlm.nih.gov/protein/XP_002007566.1?report=genbank&log$=prottop&blast_rank=27&RID=08MCBZ8W016) | 117 |
| [*Drosophila navojoa*](https://www.ncbi.nlm.nih.gov/Taxonomy/Browser/wwwtax.cgi?id=7232) | [XP_017956283](https://www.ncbi.nlm.nih.gov/protein/XP_017956283.1?report=genbank&log$=prottop&blast_rank=28&RID=08MCBZ8W016) | 117 |
| [*Drosophila hydei*](https://www.ncbi.nlm.nih.gov/Taxonomy/Browser/wwwtax.cgi?id=7224) | [XP_023169885](https://www.ncbi.nlm.nih.gov/protein/XP_023169885.1?report=genbank&log$=prottop&blast_rank=30&RID=08MCBZ8W016) | 117 |
| [*Drosophila novamexicana*](https://www.ncbi.nlm.nih.gov/Taxonomy/Browser/wwwtax.cgi?id=47314) | [XP_030570409](https://www.ncbi.nlm.nih.gov/protein/XP_030570409.1?report=genbank&log$=prottop&blast_rank=31&RID=08MCBZ8W016) | 117 |
| [*Drosophila virilis*](https://www.ncbi.nlm.nih.gov/Taxonomy/Browser/wwwtax.cgi?id=7244) | [XP_002047114](https://www.ncbi.nlm.nih.gov/protein/XP_002047114.1?report=genbank&log$=prottop&blast_rank=32&RID=08MCBZ8W016) | 117 |
| [*Drosophila grimshawi*](https://www.ncbi.nlm.nih.gov/Taxonomy/Browser/wwwtax.cgi?id=7222) | [XP_001983728](https://www.ncbi.nlm.nih.gov/protein/XP_001983728.1?report=genbank&log$=prottop&blast_rank=33&RID=08MCBZ8W016) | 117 |
| [*Drosophila albomicans*](https://www.ncbi.nlm.nih.gov/Taxonomy/Browser/wwwtax.cgi?id=7291) | [XP_034106340](https://www.ncbi.nlm.nih.gov/protein/XP_034106340.1?report=genbank&log$=prottop&blast_rank=29&RID=08MCBZ8W016) | 118 |

***Anopheles* CG6709 orthologs**

| Species | Accession number | Length (aa) |
| --- | --- | --- |
| [*Anopheles albimanus*](https://www.ncbi.nlm.nih.gov/Taxonomy/Browser/wwwtax.cgi?id=7167) | XP_035786950 | 115 |
| *Anopheles arabiensis* | A0A182I156 ^b^ | 115 |
| *Anopheles coluzzii* | A0A182LLJ5^b^ | 115 |
| *Anopheles gambiae* | XP_556944 | 115 |
| *Anopheles melas* | A0A182U894 ^b^ | 115 |
| *Anopheles merus* | A0A182VJI9^b^ | 115 |
| *Anopheles quadriannulatus* | A0A182X8L6 ^b^ | 115 |
| *Anopheles christyi* | A0A182K2B2 ^b^ | 113 |
| *Anopheles darlingi* | ADMH02000262 ^a^ | 113 |
| *Anopheles epiroticus* | A0A182PJW3^b^ | 113 |
| *Anopheles atroparvus* | A0A182JLB8 ^b^ | 112 |
| *Anopheles sinensis* | KFB36011 | 112 |
| *Anopheles culicifacies* | A0A182MX02 ^b^ | 111 |
| *Anopheles dirus* | [A0A182NH20](http://www.uniprot.org/uniprot/A0A182NH20)^b^ | 111 |
| *Anopheles farauti* | A0A182QBX7 ^b^ | 111 |
| *Anopheles funestus* | A0A182RDF1^b^ | 111 |
| *Anopheles minimus* | A0A182WCK2 ^b^ | 111 |
| *Anopheles stephensi* | [XP_035905953](https://www.ncbi.nlm.nih.gov/protein/XP_035905953.1?report=genbank&log$=prottop&blast_rank=5&RID=086SRR7P016) | 111 |

Notes: ^a^ – WGS (whole genome shotgun); ^b^ – UniProt.
